# Remarkable Alteration in the Structure of the Patella

**Published:** 1829-04-01

**Authors:** 


XLVII.

REMARKABLE ALTERATION IN THE STRUCTURE OF THE PATELLA. By R. KNOX, M.D.*

A female subject was brought into Dr. Knox's dissecting-rooms, seemingly about 30 years of age, and "*who had been in good condition at the time of death,*" (one of Burke's, perhaps) whose knee-joint, when cut into, presented the following appearances.

In the left knee was a loose cartilage, similar to those which are usually described, lying firmly impacted behind the internal condyle of the femur, in a situation from which it could not easily have moved, and where it appeared to have been for a considerable length of time. It was bound down in this situation in a remarkable manner by the synovial capsule, and the cartilage of the condyle was rough, villous, soft, in fact, almost gone. The trochlea of the femur, on which the patella usually plays, was somewhat changed in form; both condyles superiorly and lightly overlapped the femur, and the smooth exterior surface of the external condyle was becoming converted into an ivory-looking substance, whilst its cartilage was likewise scarcely perceptible. Near the insertion of the anterior crucial ligament, was a small exostosis from the tibia, but the most remarkable appearance was that presented by the patella.

"Developed, apparently, in the tendon of the extensor, and surmounting the upper margin of the patella, there is an additional bone, as if a second patella The remaining portions are three; a large central portion, corresponding to the regular patella, whose inner or articular surface is evidently undergoing the same change as that surface of the femur on which it must chiefly have played, namely, the outer condyle: and on the margins of this patella, viz. on the internal and inferior, but, at the same time, somewhat external margins, there are two additional portions of bone, of considerable magnitude, organized, apparently, altogether as the original patella, and performing similar functions.

"The preparation might easily have been taken by any one for a specimen of fractured patella, were it not for two circumstances—the presence of loose cartilarge in the joint, and the fact, that the other knee of the same subject presents altogether the same appearances, so that whatever I have said of the left knee is strictly applicable to the right, and *vice versâ.* I do not remember ever to have seen such a pathological specimen, nor do I remember to have read of such.

"In the extensive museum entrusted to my charge by the Royal College of Surgeons, there is a preparation, exhibiting a loose cartilage in the joint, altogether like the cartilaginous bodies now before us, and a commencement of that change on the margin of the condyle, which I have described as an overlapping of the shaft of the bone by a projection of condyle; but the patella has not undergone any change; still I am inclined to think, that nearly all the pathological appearances I have described were produced by the loose cartilage, and by the circumstance of its being impacted in the upper and back part of the joint, so as to prevent the proper extension of the limb."

Whatever the cause of this singular condition of the patella might have been, it is remarkable that *both* the knee-joints should have been affected. Such cases as these are not of much value, practically speaking, in themselves, but they grow into importance, from affording information and aiding diagnosis in future affections of a similar nature. For this reason we have placed this pathological fact on record in our pages.

---

* Edinb. Med.-Chir. Trans. vol. III. part I.

